# Verification of a cryptic t(Y;15) translocation in a male with an apparent 45,X karyotype

**DOI:** 10.1186/s13039-022-00581-6

**Published:** 2022-02-14

**Authors:** Shengfang Qin, Xueyan Wang, Jin Wang, Zhuo Zhang, Ximin Chen, Yan Yin, Mengling Ye, Jesse Li-Ling

**Affiliations:** 1 Department of Medical Genetics and Prenatal Diagnosis, Sichuan Provincial Maternity and Child Health Care Hospital, Chengdu, 610045 Sichuan China; 2 Department of Medical Genetics, West China Second Hospital, Sichuan University, Chengdu, 610041 Sichuan China

**Keywords:** 45,X male, Y chromosome translocation, Sex-determining region Y gene, Azoospermia factor, Fluorescence in situ hybridization, Chromosomal microarray analysis

## Abstract

**Background:**

A rare disease is that an individual with a non-chimeric karyotype of 45,X develops into a male. We explored the genetic aetiology of an infertile male with an apparent 45,X karyotype, which was subsequently verified as cryptic translocation between chromosomes Y and 15.

**Methods:**

DNA was extracted from the patient's peripheral blood. A range of genetic testing was performed, including conventional chromosomal karyotyping, short tandem repeat (STR) analysis for azoospermia factor (*AZF*) region, fluorescence in situ hybridization (FISH) with specific probes groups of *DXZ1/DYZ3*, *DYZ3*/*D15Z1/PML* and *SRY/D15Z1/PML*, and chromosomal microarray analysis (CMA) for genomic copy number variations (CNVs).

**Results:**

The patient was found to have an apparent 45,X karyotype. STR analysis showed that he possessed a short arm of the Y chromosome, including the *SRY* gene; however, he was missing the long arm of the Y chromosome, including *AZF*a + b + c and Yqter. A FISH assay of *DXZ1* and *DYZ3* probes showed a green signal of the X centromere and a red of the Y centromeric signal on a D-group-sized chromosome. By FISH assaying with *D15Z1* and *DYZ3* probes, chromosomes 15 and Y centromeric signals appeared closely on a single chromosome, as the *PML* control probe ascertained. A further FISH assay with *D15Z1* and *SRY* probes revealed a signal of the *SRY* gene at the end of one arm of chromosome 15. The result of the CMA indicated a deletion with an approximate size of 45.31 Mb spanning from Yq11 to Yter.

**Conclusion:**

Our study enriched the karyotype-phenotype correlation of Y and 15 chromosomes translocation. It strengthened the critical roles of molecular genetic techniques in identifying the chromosomal breakpoints and regions involved. Genetic aetiology can guide early intervention in childhood and assisted reproduction in adulthood.

## Background

Most individuals with a 45,X karyotype will develop into females with a Turner syndrome phenotype. However, very rare 45,X individuals are sterile males with testes. So far, fewer than 40 cases of 45,X males have been reported, and most of them have harboured chimeric XY cells [1, 2]. Only about 10 cases were discovered as 45,X males who do not possess the Y chromosome but translocation of the Y chromosome with an autosome [3–5]. The translocation rate of the Y chromosome and an autosomal is low to 1/2000 [5]. The Y chromosome harbours genes essential for testis development and function, such as the master gene for testis determination (*SRY*) and the genes residing in the azoospermia factor (*AZF*) regions. So, it is the most critical molecular genetic basis in male gender determination and fertility [6, 7]. There are two types of consequences of Y/autosome translocations: Individuals with balanced translocations usually have no abnormal clinical feature. However, Unbalanced translocations may have different clinical manifestations according to increased or decreased genetic material or damaged genes. For example, *AZF* region deletion of the Y chromosome is often presented as azoospermia and infertility [8]. Of note, about 70% of translocations between the Y and a telocentric chromosome involve chromosome 15, which may be attributable to the homology between heterochromatin sequences at 15p and Yq [9]. Furthermore, t(Y;15) is usually unbalanced, with most breakpoints occurring on 15p (15p11-13) and the heterochromatin region of Yq12. We hereby report on a male with an apparent 45,X karyotype, which was subsequently verified as cryptic translocation between chromosomes Y and 15.

## Methods

### Subject

The patient, a 27-year-old male, was referred to our hospital due to primary infertility. With a height of 166 cm (−0.5SD) and a weight of 51.5 kg (−1.2SD), the patient had a male appearance with a few whiskers and Adam’s apple. He was found to have a small testis measured approximately 8 mL on both sides. Sperms were not found on three routine semen examinations. The levels of sex hormones examined at another hospital were as follows: testosterone: 15.12 nmol/L (reference value: 4.94–32.01 nmol/L), prolactin: 168.09 nmol/L (reference value: 77.75–435.92 nmol/L), estradiol: 70.4 pmol/L (reference value: 40.4–161.5 pmol/L), luteinizing hormone: 5.33 IU/L (reference value: 0.57–12.07 IU/L), follicle-stimulating hormone 13.24 IU/L (reference value: 0.95–11.95 IU/L). His father and mother denied a family history of genetic disorders and consanguinity. No infertility problems or similar patients existed in their relatives. According to the recollection of the patient and his parents, the patient had no apparent abnormalities in the process of growth and development compared with other boys of the same age. He had never been had a growth hormone and sex hormone test until he came to our hospital. The karyotypes of his father and mother were 46, XY and 46, XX, respectively.

### Specimen preparation

Peripheral blood samples of the patient and his parents were collected with heparin sodium and EDTA-Na_2_ anticoagulant tubes, respectively.

### Chromosomal karyotyping analysis

Lymphocytes from heparin sodium anticoagulated blood were cultured, harvested, and loaded onto microscope slides for Giemsa staining using conventional methods. As previously described, a Zeiss (Germany) karyotype analysis system was adopted for chromosome counting and karyotype analysis [10]. Chromosome interpreted according to International System of Human Cytogenetic Nomenclature (ISCN) criteria [11].

### DNA extraction

Genomic DNA was extracted from EDTA-Na_2_ anticoagulated blood with a QIAamp DNA Mini Kit (QIAGEN, Germany) by following the manufacturer’s instructions. DNA was qualified with a concentration over 30 ng/μL and an OD_260_/_280_ value between 1.8 to 2.0, as determined by ultraviolet spectrophotometer Nanodrop 1C (Thermo Fisher Scientific, USA).

### Analysis of *AZF* sequences

Y chromosome-specific sequences were detected using the short tandem repeats (STR) method. The PCR conditions were as follows: 94 °C for 2 min, 98 °C 10 s, 60 °C 30 s, 68 °C 30 s, 25 cycles; 72 °C for 10 min. The amplicons were subjected to capillary electrophoresis on an ABI 3500Dx gene analyzer. Then, the data were analyzed by using GeneMapper software. These specific STR loci are selected mainly based on sex chromosome ploidy and *AZF* microdeletion analysis. Sex chromosome ploidy was obtained by analyzing these STR loci. The STR loci of *ZFX* and *ZFY* are used to detect the number of X and Y chromosomes; Yqp to measure the ratio of Yq and Yp, Xqp to measure the ratio of Xq and Xp; C03Yp and C03Xq to detect the copy number of Yp and Xq, taking chromosome 3 as a reference. *AZF* microdeletion was informed through detecting the classical loci of sY84, sY86, sY127, sY134, sY254, sY255 of the *AZF* region in Yq. The *TAF9b* gene located on chromosome 3 is highly conserved, so taking it as a reference, the X chromosome can be accurately counted. The absence of the Y chromosome can be judged compared to the X chromosome.

### Fluorescence in situ hybridization (FISH) analysis

#### FISH assay with *D18Z1*, *DXZ1* and *DYZ3* probes

Metaphase cells derived from cultured peripheral blood lymphocytes were hybridized with *D18Z1, DXZ1* and *DYZ3* probes (China Medical Technologies, Inc. Beijing, China), which targeted the DNA stretches of 18, X, and Y centromeres, respectively. Glass slides were denatured at 78 °C for 10 min and hybridized at 42 °C for more than 16 h. After that, the signals were observed under a fluorescence microscope.

#### FISH assay with *PML*, *D15Z1* and *DYZ3* probes

Metaphase cells loaded upon glass slides were hybridized with the *PML*, *D15Z1* and *DYZ3* probes. The hybridization buffer and the *DYZ3* probe were mixed with a proportion of 4:1 and applied to the cell-loaded glass slides. Denaturation and hybridization were carried out by following the standard procedure.

#### FISH assay with *PML*, *D15Z1* and *SRY* probes

The procedure was the same as the prior step, only differed with the preparation of the hybridization mix, in which the buffer and the *SRY* probe were mixed at a proportion of 9:1.

### Chromosomal microarray analysis (CMA)

500–1000 μg of genomic DNA and the same amount of reference DNA were used for the experiment. After digestion, the labelled patient sample was mixed with the reference and hybridized to a SurePrint G3 CGH + SNP (180 K) chip. Fluorescence signals were scanned with an Agilent DNA Microarray Scanner. Data were extracted from the chip image with Agilent Feature Extraction Software and converted into log-ratios. Copy number variants (CNVs) were analyzed with Agilent CytoGenomics Software (Agilent Technologies, USA). Candidate variants were queried with relevant online databases such as OMIM (https://omim.org/), DGV (http://dgv.tcag.ca/dgv/), Decipher (https://decipher.sanger.ac.uk/), ClinGen (https://www.clinicalgenome.org/) and ClinVar (https://www.ncbi.nlm.nih.gov/clinvar/).

### Comparison of the clinical phenotypes of patients with a 45,X,t (Y;15) karyotype

Cases with 45,X,dic(Y;15) karyotype has been searched for in the previous report, and their clinical phenotypes were compared.

## Results

### Cytogenetic analysis

**Fig. 1:**
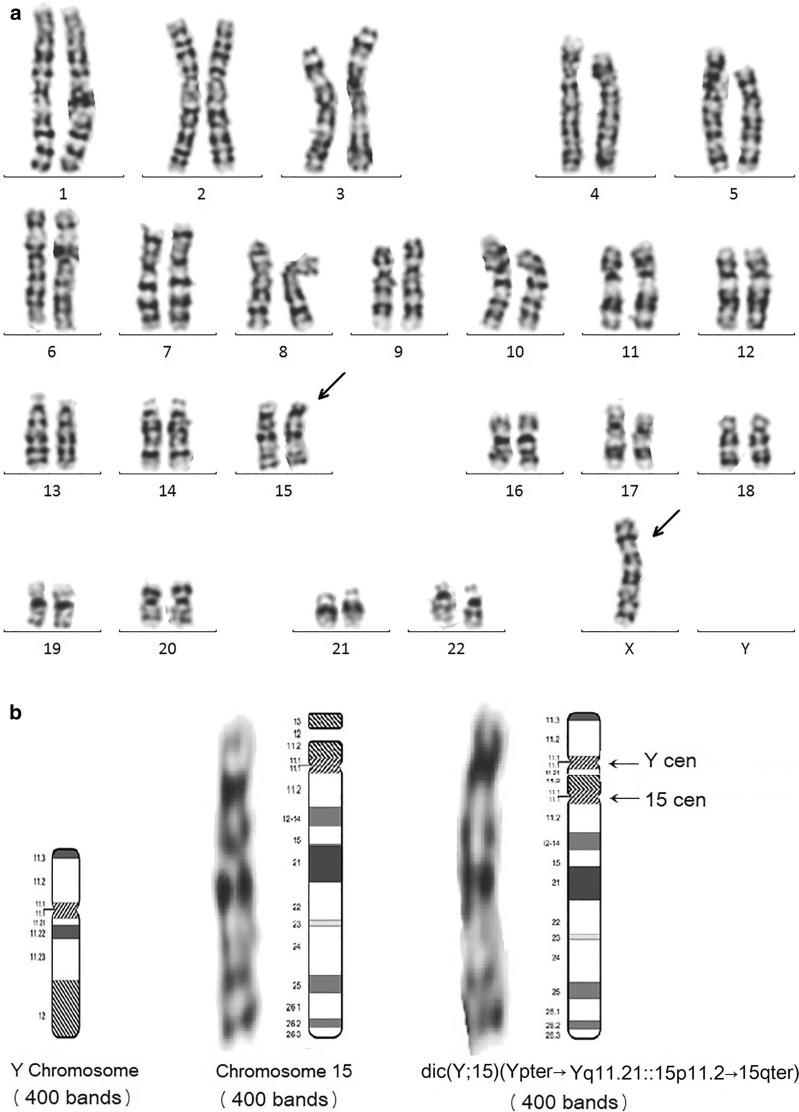

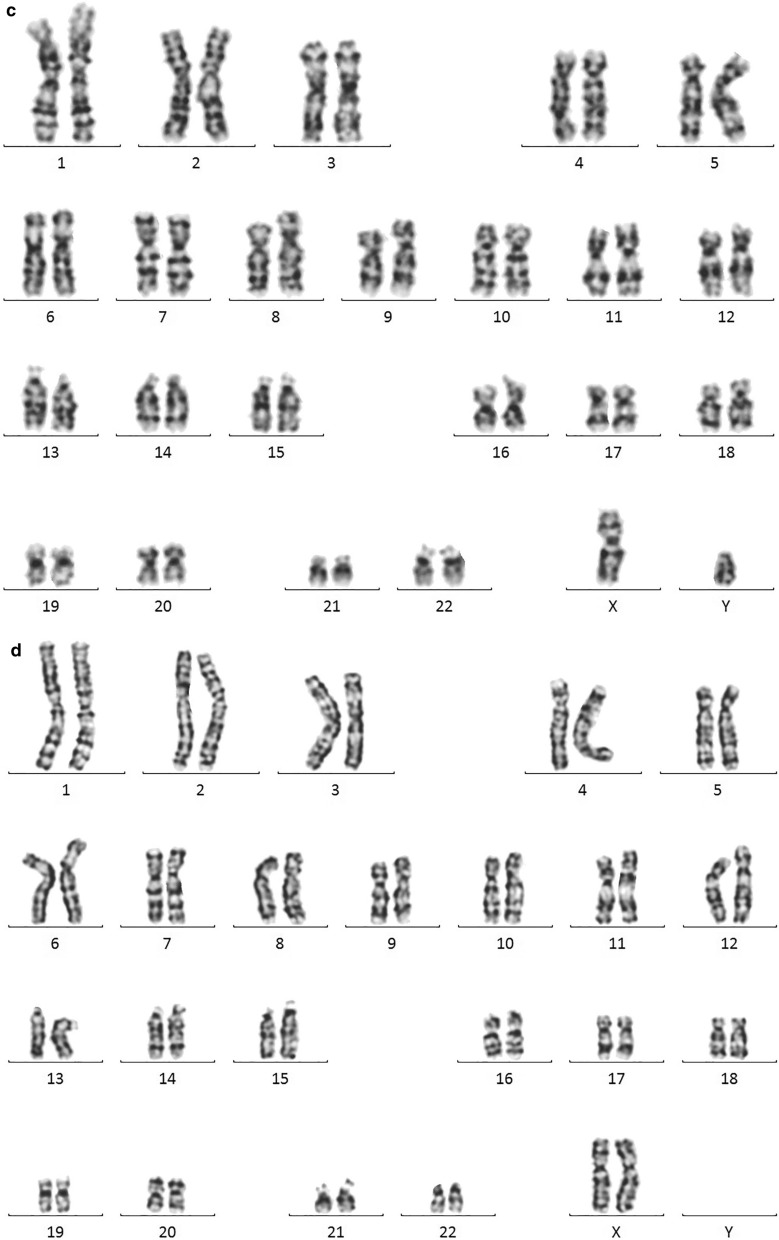
**a**: Karyogram of the t(Y;15) male patient. The der(15) and X chromosomes are indicated by arrows.** b**: Ideograms of chromosomes Y, 15, and dic(Y;15). The Y centromere and 15 centromere of the dicentric chromosome are indicated by arrows.** c**: 46,XY karyogram of the patient's father.** d**: 46,XX karyogram of the patient's mother

The patient’s father and mother had a karyotype of 46,XY and 46,XX, respectively. In contrast, the patient had an apparent 45,X karyotype (Fig. 1).

### STR analysis

**Fig. 2:**
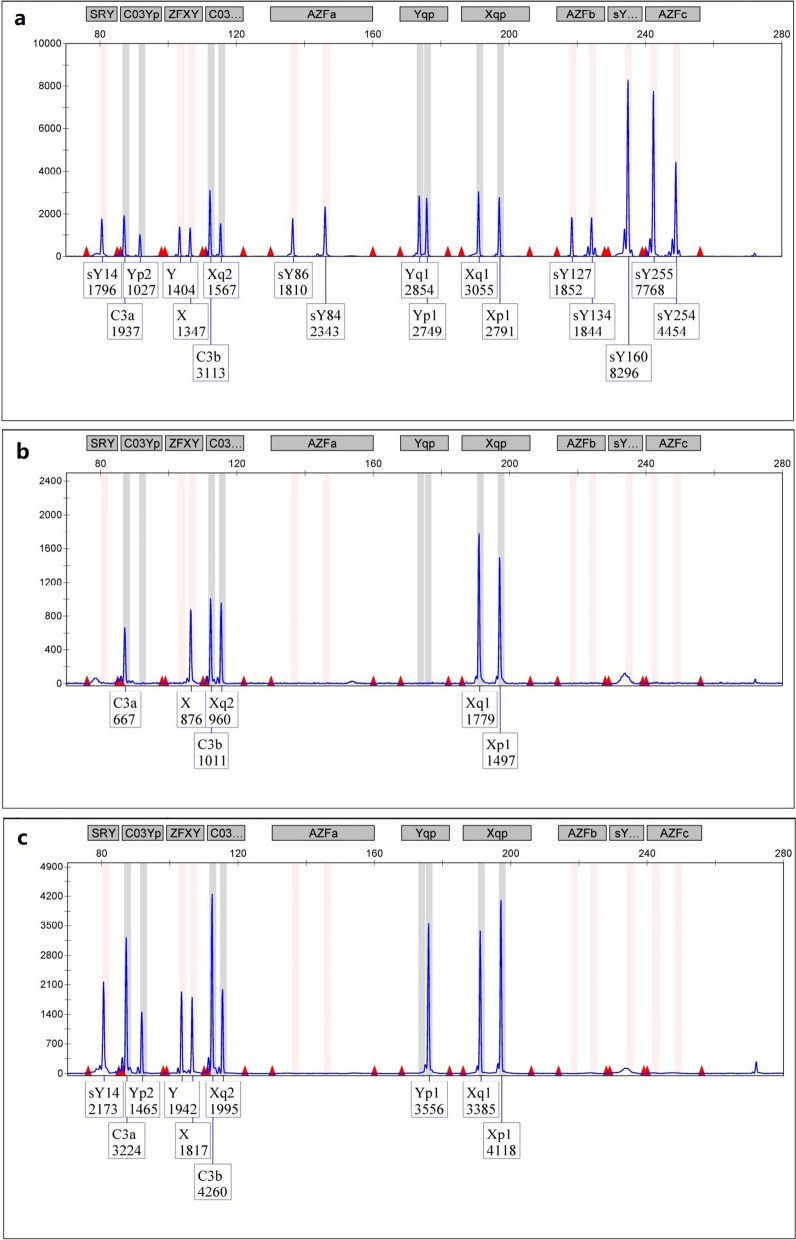
Capillary electrophoresis diagram for the detection of *AZF* sequences. **a**: Normal male; **b**: Normal female; **c**: 45,X male. A fluorescence peak representing the *SRY* sequence was seen, but those for the *AZF*a, *AZF*b, *AZF*c, and SY160 were absent, suggesting loss of the whole long arm of the Y chromosome

STR analysis showed that the patient was positive for the *SRY* gene mapped to Yp but negative for the *AZF*a + b + c and Yqter sequences of Yq. He had only possessed a single copy of Xp, Xq, and Yp (Fig. 2).

### FISH analysis

#### FISH with the *D18Z1*, *DXZ1* and *DYZ3* probes

**Fig. 3:**
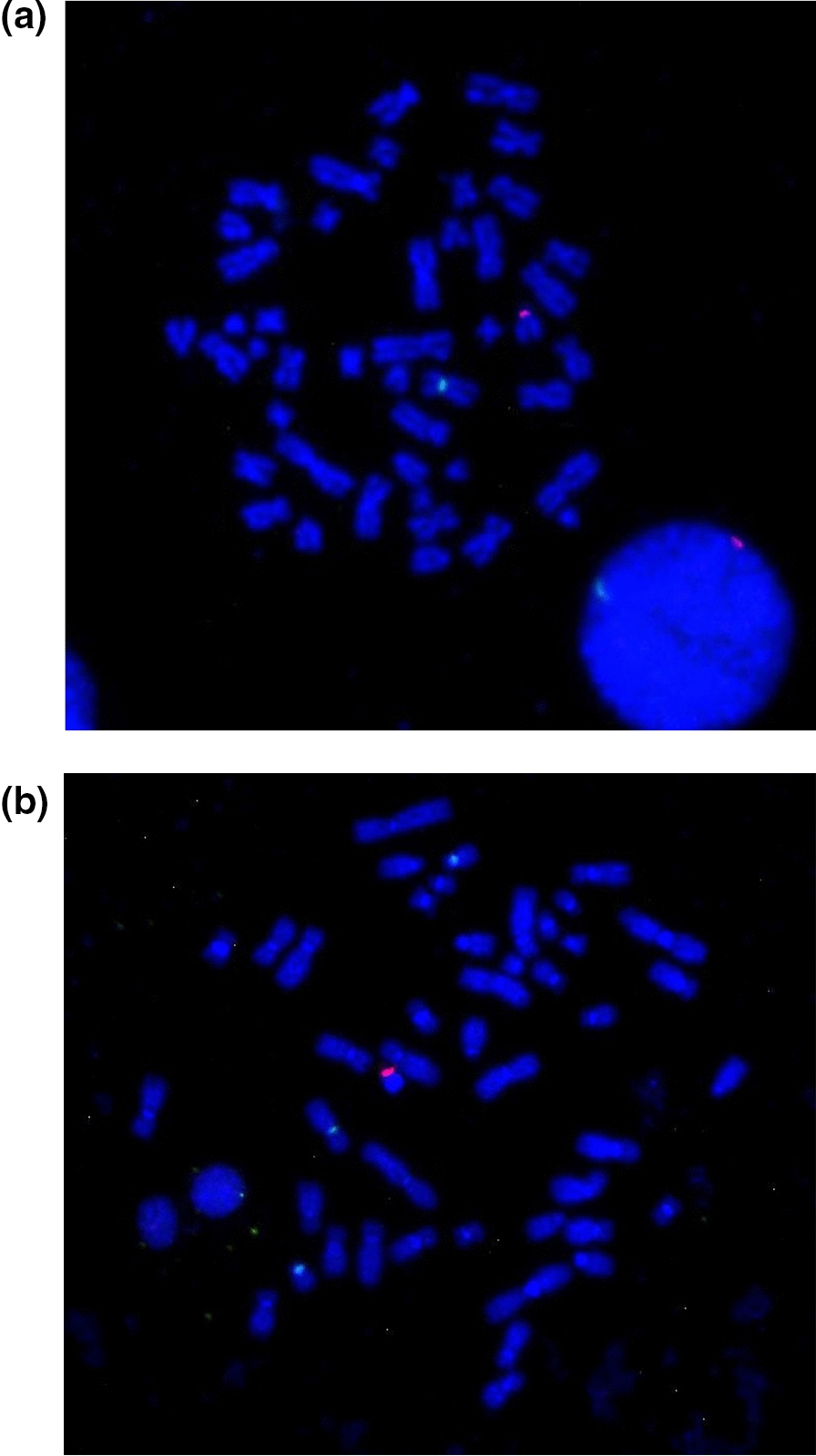
**a**: FISH image of metaphase cells of the patient detected with the X and Y centromeric probes. The green signal indicated the centromere of the X chromosome, while the red signal indicated the Y chromosome material on a D-group-telocentromeric chromosome; **b**: FISH image of metaphase cells of the patient's father detected with the 18, X and Y centromeric probes. The blue, green, red signals are indicated the 18, X, Y chromosomes

FISH with *D18Z1/DXZ1/DYZ3* centromeric probes showed two blue, one green, and one red signal, respectively. However, the red signal of the Y centromere was observed on one of the D-group-sized telochromosomes in the patient’s metaphase cells (Fig. 3).

#### FISH with the *PML*, *D15Z1* and *DYZ3* probes

**Fig. 4:**
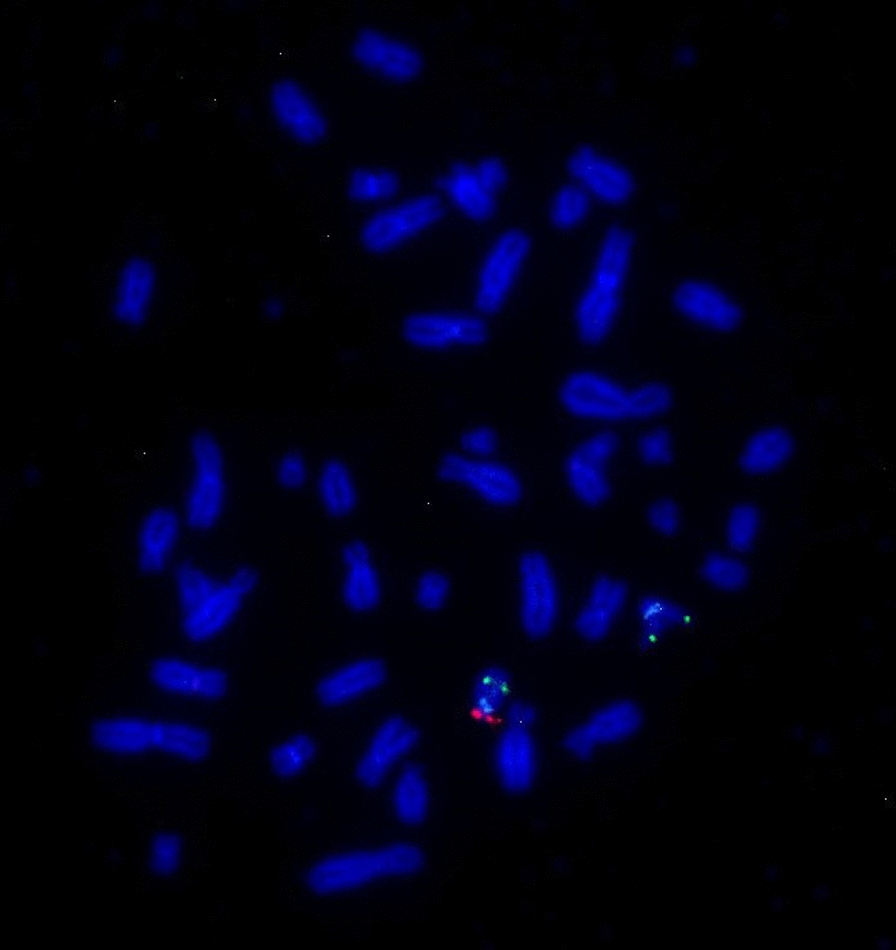
FISH image of metaphase cells of the patient detected with centromeric probes for chromosomes 15 and Y. The green signal is from the *PML* probe mapped to 15q24.1, the red signal is from the *DYZ3* probe mapped to the centromere of Y, and the aqua blue signal is from the *D15Z1* probe (15q10) closely located on the same chromosome

FISH with the *PML*, *D15Z1* and *DYZ3* probes has revealed one green, one aqua blue, and one red signal in one of the D-group-sized telochromosomes, respectively. The aqua blue and red signals of *D15Z1* and *DYZ3* were in close proximity, which indicated that chromosome 15 of the patient was dicentric and has contained materials from chromosomes 15 and Y (Fig. 4).

#### FISH with the *PML*,* D15Z1* and *SRY* probes

**Fig. 5:**
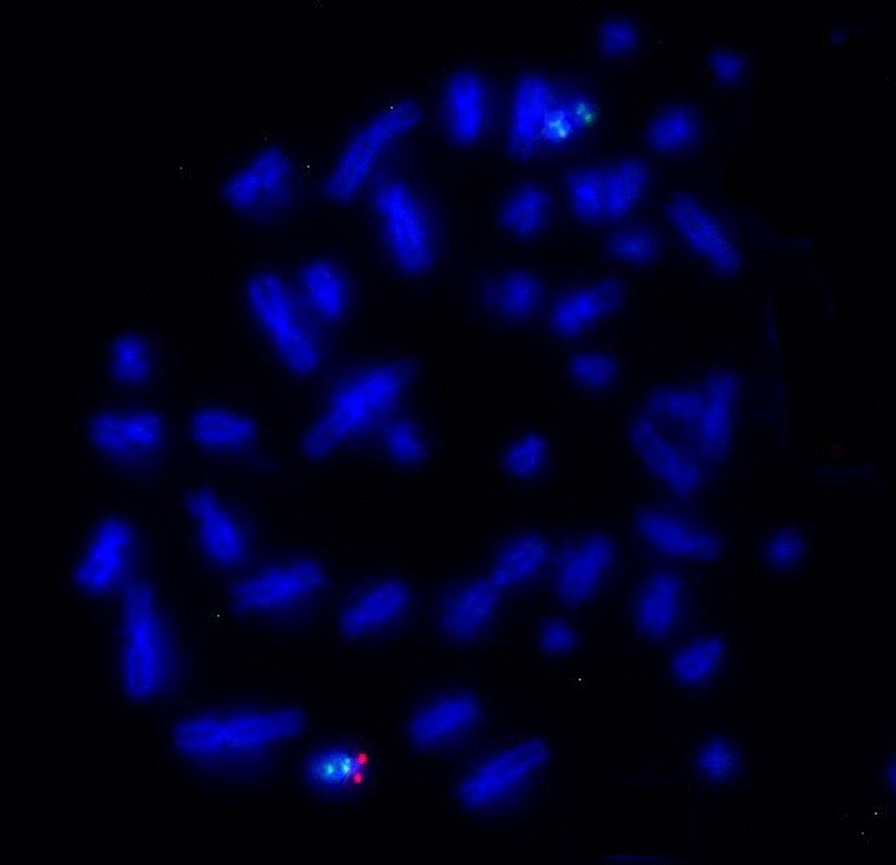
FISH image of metaphase cells of the patient detected with probes for chromosome 15 and the *SRY* region. The green signal is from the *PML* probe mapped to 15q24.1, the orange signal from the *SRY* probe (Yp11.31) and the aqua blue signal from the *D15Z1* probe (15q10) are located on the same chromosome

A further FISH assay with the *PML*, *D15Z1* and *SRY* probes revealed fluorescence signals for all three probes on the same telocentromeric chromosome, including an aqua blue signal of *D15Z1*, a green signal of *PML* on chromosome 15, and an orange signal of *SRY*. The *SRY* probe signal was observed on the opposite arm of the *PML* probe at 15q24.1, which suggested the *SRY* gene has translocated to 15p (Fig. 5).

### Result of CMA analysis

**Fig. 6:**
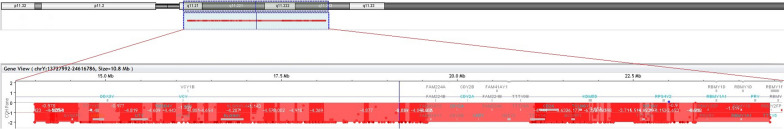
CMA result of the patient. The Yq region was absent in the patient. The red bar indicates the area of deletion

The CMA result of the patient was arr[GRCh37] Yq11.21q11.23(13988156_59301502) × 0, i.e. 45.31 Mb (Fig. 6). We postulated the deletion range from Yq11.21 to Yqter because the microarray chip contained no probe for the heterochromatin region from Yq11.23 to Yqter. The above result was also in keeping with the STR analysis, which showed no peaks for the *AZF* sequences at Yq and the *SY160* sequence at Yqter.

### Comparison of the clinical phenotypes of individuals with a 45,X,dic(Y;15) karyotype

Table 1 has summarized the clinical features of individuals with a 45,X,dic(Y;15) karyotype as shown; variation of the breakpoints has resulted in discrepancies in the deletion regions on chromosomes Y and 15. Patients 2, 3, and 4 had lost no genetic material; therefore, they had no abnormal phenotypes. Patient 5 showed severe oligoasthenospermia due to partial deletion of *AZF*c (sY254). Patients 6 and 7 had lost the entire long arm of the Y chromosome (including the *AZF*a + b + c regions), similar to our patient. They showed abnormal phenotypes, such as spermatogenous and testicular dysplasia.

**Table 1:** Summary of chromosomal breakpoints and clinical phenotypes of individuals with a 45,X,dic(Y;15) karyotype

Case	References	Chromosomal karyotype	Clinical phenotype	Analytical method	Whether the derivative chromosome contains the Y centromere	Whether the derivative chromosome contains the 15 centromere
1	Present study	45,X,dic(Y;15)(q11;p11).ish dic(Y;15)(*SRY* + ,DYZ3 + ;D15Z1 + ,PML +)	A 27-years-old male, with a weight 51.5 kg and a height of 160 cm, had small testes. Laboratory tests found normal level of testosterone, high level of follicle-stimulating hormone. He had azoospermia due to deletion of *AZFa* + *b* + *c* loci, but had a 45,X karyotype	Karyotyping, FISH, Multiplex PCR, CMA	Yes	Yes
2	Subrt et al. [15]	45,X,t(Y;15)(Yqter → Yp11::15q11 → 15qter)	Four males from four consecutive generations of a pedigree harbored 45,X,t(Y;15) translocations but with a normal phenotype	Karyotyping	Yes	No
3	Mahmut [18]	45,X,t(Y;15)(q12;q11)	The karyotypes of father and mother were 46,XY, t(15;20)(q11; 13) and 46,XX, respectively, but the fetus was a 45,X,t(Y;15) male, and no abnormal phenotype was observed up to one year after birth	Karyotyping, FISH	Yes	No
4	White et al. [19]	45,X,dic(Y;15)(q11.23;p11.1)	The karyotype of the fetus was the same as that of the father, and no abnormal phenotype was observed	Karyotyping, FISH Microsatellite analysis	Yes	Yes
5	Lin et al. [22]	45,X, der(15)(?::p11.2 → qter)dn. ish psu dic(Y;15)(q12;p11.2)(*D15Z1* + , *SNRPN* + , *PML* + ; *SRY* + ,*DYZ3* + , *DYZ1* +)	A 33-year-old male had normal intelligence, growth and development, testicular size and sex hormones level but infertility. He had severe oligoasthenospermia due to partial *AZFc* (sY254) deletion	Karyotyping, FISH, Multiplex PCR	Yes	Yes
6	Antonio et al. [8]	45,X,der(15)(Ypter → q22.21::15p11.2 → qter)	A 41-year-old male, 58 kg in weight and 157 cm in height, had small testis, epididymis dystrophy. Laboratory tests found low testosterone, high gonadotropin, azoospermia, and deletion of *AZFa* + *b* + *c* loci	Karyotyping, FISH, Y microdeletion analysis	Yes	Yes
7	Schempp [23]	45,X,t(Y;15)(p10;p12)	A 19-year-old male had a weight of 54 kg and a height of 154 cm. He had normal mental development and no deformity. He had a de novo translocation between chromosomes Y and 15. His primary anomaly is azoospermia	Different chromosome staining	Yes	Yes

## Discussion

Among the previously reported 45,X,t(Y;15) male cases, only a few were non-chimeric. Our patient's results showed no other cells except for 45,X after analyzing two hundred cultured lymphocyte cells with karyotype and FISH methods as previous reports [1, 2]. Meanwhile, metaphase cells were analyzed with FISH probes of chromosome Y and chromosome 15. They showed the translocation of Yp onto one of 15 chromosomes. Some were initially detected with a 15p + karyotype but later confirmed as 45,X,t(Y;15) by molecular methods [12–14]. Of note, some of these translocations were inherited from parents with a normal phenotype [15–17]. As summarized by Table 1, variation of the breakpoint sites in these 45,X,t(Y;15) males has resulted in their phenotypes' heterogeneity. The individual with a loss of heterochromatin had a normal phenotype [15, 18, 19]. By contrast, individuals with loss of euchromatin had various clinical phenotypes. Loss of the *AZF*c region at Yq presented severe oligoasthenospermia [20], while the *AZF*a, *AZF*b and *AZF*c regions manifested azoospermia, testicular dysplasia and other phenotypes [8, 21].

The contribution of genetic testing performed here was different for the patient's diagnosis. Karyotyping was used to count chromosomes and assess chimaera. CMA analyses were adopted to map the deletion region accurately and exclude deletion and duplication of other genomic regions. STR was proper for *AZF* region microdeletions. FISH was helpful for the breakpoint location of the Y-15 chromosomal translocation. The cause of the patient's azoospermia has been ultimately verified. It was attributable to the unbalanced translocation between chromosomes 15 and Y, resulting in the deletion of the *AZF*a + b + c region on Yq. Therefore, combining these genetic techniques is our recommendation for clinicians to diagnose similar patients.

In the present study, the patient appeared to have no Y chromosome but was found to carry an unbalanced Y → 15 translocation by molecular genetic testing. The translocation has resulted in the *SRY* gene of Yp exchange onto the short arm of chromosome 15. Two broken chromosomes containing the centromeric parts have formed a dicentric aberration. Dicentric chromosomes derived from translocations between chromosomes Y and 15 are rare [22]. As one of the centromeres was inactive or nonfunctional, the dicentric chromosome might behave and segregate as a monocentric chromosome during cell division [23, 24]. A translocation study between the Y and chromosome 21 found that the Y chromosome's centromere was preferentially inactivated in pseudodicrocentromeres [25]. However, it is not sure which centromere is active because we have not directly measured the activity. Moreover, it is not apparent from the morphology of the centromeres. The acentric fragments, including the short arm of chromosome 15 and the long arm of chromosome Y, are prone to lose during subsequent cell divisions. As a result, only 45 chromosomes were left. Cytogenetically, the derivative chromosome 15 containing a small fraction of Yp could not be easily distinguished from the normal ones.

The heterochromatin of 15pter should be lost when Yp translocates to 15p. However, the CMA has no probes assigned in the telocentric satellite region. The results of CMA analysis showed no loss of chromosome 15 genetic material. Several males carrying a Y-acrocentric chromosome translocation with a breakpoint between Yq11 and Yq12 were reported previously [26, 27]. In the present study, based on the results of molecular analysis, our patient's karyotype was verified as 45,X,dic(Y;15)(q11;p11).ish dic(Y;15)(*SRY*+ ,*DYZ3*+ ;*D15Z1*+ ,*PML*+). Results of the FISH assay indicated that the patient's chromosomal rearrangement had occurred de novo, as no abnormality was found with his father. It may be postulated that the two chromosomes had broken during the first meiosis of spermatogenesis or at a very early stage of zygote formation. Human embryos will develop towards the male gender as long as the *SRY* gene is present in the genome, even without the Y chromosome. That may also be the primary molecular basis for 45,X males [28, 29]. Based on molecular testing, the seemingly "pure" 45,X male may not exist. The *SRY* gene on autosomes derived from the Yp translocation could explain the male sex determination in such cases [30].

Similar to Hsu et al*.* report [30], the main clinical features of our patient were azoospermia and infertility. Several genes mapped to the *AZF* region of Yq, including *USP9Y*, *DBY*, *PRY*, *RBMY*, *DAZ* and *BPY2*, are involved in the formation, development, and maturation of sperms. Deletions of the *AZF* region have been the most common risk factor for male infertility [8, 31, 32]. Account for 10–15% of azoospermia and 5–10% of severe oligozoospermia [33]. The type and location of the *AZF* gene deletions are correlated with the severity of fertility disorders. *AZF*a deletion usually results in SCOS-only type I syndrome (SCOS type I) and azoospermia. *AZF*b deletion is associated with azoospermia caused by the cessation of meiosis. *AZF*c deletion has considerable clinical heterogeneity [8]. In the present study, the patient has lost Yq (including *AZF*a + b + c), and no sperm was found upon routine semen tests. Microspermatocentesis was not recommended for this patient because his testis did not produce any sperm as previous report [34]. He has no chance of having a biological child, so this translocation will not be passed on.

Infertile reasons for this 45,X,t(Y;15) male patient may be mainly attributed to the loss of spermatogenetic genes in the *AZF* region and the disability of the critical step of the sex-chromosomal pairing during the meiotic prophase [35–37]. We speculated that the failure of X–Y pairing led to germ cell loss because of the loss of the YPAR2 region and the translocation of the YPAR1 region.

Our study enriched the karyotype-phenotype correlation of Y and 15 chromosomes translocation and strengthened the critical roles of molecular genetic techniques in identifying the chromosomal breakpoints and regions involved. Early diagnosis can guide their clinical intervention by correcting the external genitalia, removing gonad dysplasia, hormone therapy, and assisted reproduction.

## Data Availability

The datasets used and/or analysed during the current study are available from the corresponding author on reasonable request.

## References

[1] Forabosco A, Carratu A, Assuma M, De Pol A, Dutrillaux B, Cheli E (1977). Male with 45, X karyotype. Clin Genet.

[2] Graham BH, Bacino CA (2003). Male patient with non-mosaic deleted Y-chromosome and clinical features of Turner syndrome. Am J Med Genet A.

[3] Bilen S, Okten A, Karaguzel G, Ikbal M, Aslan Y (2013). A 45 X male patient with 7q distal deletion and rearrangement with SRY gene translocation: a case report. Genet Couns.

[4] Turleau C, Chavin-Colin F, de Grouchy J (1980). A 45, X male with translocation of euchromatic Y chromosome material. Hum Genet.

[5] Nielsen J, Rasmussen K (1976). Y/autosomal translocations. Clin Genet.

[6] McLaren A (1990). Sex determination. What makes a man a man?. Nature.

[7] Krausz C, Casamonti E (2017). Spermatogenic failure and the Y chromosome. Hum Genet.

[8] Colaco S, Modi D (2018). Genetics of the human Y chromosome and its association with male infertility. Reprod Biol Endocrinol.

[9] Metzler-Guillemain C, Mignon C, Depetris D, Guichaoua MR, Mattei MG (1999). Bivalent 15 regularly associates with the sex vesicle in normal male meiosis. Chromosome Res.

[10] Liu Y, Kong XD, Wu QH, Li G, Song L, Sun YP (2013). Karyotype analysis in large-sample infertile couples living in Central China: a study of 14965 couples. J Assist Reprod Genet.

[11] McGowan-Jordan J, Hantings RJ, Moore S (2020). ISCN 2020: an international system for human cytogenomic nomenclature(2020).

[12] Neumann AA, Robson LG, Smith A (1992). A 15p+ variant shown to be a t(Y;15) with fluorescence in situ hybridisation. Ann Genet.

[13] Zhang LL, Lu BT, Yao J (2010). Detection of a pedigree with a 15p+ chromosomal karyotype with Y-specific probes. Zhonghua Yi Xue Yi Chuan Xue Za Zhi.

[14] Zhao L, Li H, Xue YQ, Pan JL, Wu YF, Lu M (2004). Application of fluorescence in situ hybridization in the diagnosis of genetic diseases. Zhonghua Yi Xue Yi Chuan Xue Za Zhi.

[15] Subrt I, Blehová B (1974). Robertsonian translocation between the chromosome Y and 15. Humangenetik.

[16] Chen-Shtoyerman R, Josefsberg Ben-Yehoshua S, Nissani R, Rosensaft J, Appelman Z (2012). A prevalent Y;15 translocation in the Ethiopian Beta Israel community in Israel. Cytogenet Genome Res.

[17] Chen PY, Yen JH, Cheng CF, Chen PC, Li YS, Li TY (2016). Prenatal diagnosis of the maternal derivative chromosome der(15)t(Y;15)(q12;p13) in a dizygotic twin pregnancy. Ci Ji Yi Xue Za Zhi.

[18] Yıldırım MS, Arslan AB, Zamani AG (2021). Interchromosomal effect: report of a father and son, bearing different translocations of the same chromosome, and a review of the current literature. Andrologia.

[19] White LM, Treat K, Leff A, Styers D, Mitchell M, Knoll JH (1998). Exclusion of uniparental inheritance of chromosome 15 in a fetus with a familial dicentric (Y;15) translocation. Prenat Diagn.

[20] Lin S, Xie Y, Wu J, Fang Q, Chen Z, Chen B (2014). Cytogenetic and molecular study of a patient with severe oligozoospermia and asthenozoospermia. Zhonghua Yi Xue Yi Chuan Xue Za Zhi.

[21] Mancini A, Zollino M, Leone E, Grande G, Festa R, Lecce R (2008). A case of 45, X male: genetic reevaluation and hormonal and metabolic follow-up in adult age. Fertil Steril.

[22] Gal A, Weber B, Neri G, Serra A, Müller U, Schempp W (1987). A 45, X male with Y-specific DNA translocated onto chromosome 15. Am J Hum Genet.

[23] Earnshaw WC, Migeon BR (1985). Three related centromere proteins are absent from the inactive centromere of a stable isodicentric chromosome. Chromosoma.

[24] Sullivan BA, Schwartz S (1995). Identification of centromeric antigens in dicentric Robertsonian translocations: CENP-C and CENP-E are necessary components of functional centromeres. Hum Mol Genet.

[25] Fisher AM, Al-Gazali L, Pramathan T, Quaife R, Cockwell AE, Barber JC (1997). Centromeric inactivation in a dicentric human Y;21 translocation chromosome. Chromosoma.

[26] Rosa MD, Brasi DD, Zarrilli S, Paesano L, Pivonello R, Agostino A (1997). Short stature and azoospermia in a patient with Y chromosome long arm deletion. J Endocrinol Invest.

[27] Alitalo T, Tiihonen J, Hakola P, de la Chapelle A (1988). Molecular characterization of a Y;15 translocation segregating in a family. Hum Genet.

[28] Amaro A, Mafra FA, Valada Pane CE, Kulikowski LD, Bianco B, Barbosa CP (2015). 45, X karyotype in an infertile man: how is this possible. Urol Int.

[29] de la Chapelle A, Page DC, Brown L, Kaski U, Parvinen T, Tippett PA (1986). The origin of 45, X males. Am J Hum Genet.

[30] Hsu LY (1994). Phenotype/karyotype correlations of Y chromosome aneuploidy with emphasis on structural aberrations in postnatally diagnosed cases. Am J Med Genet.

[31] Motovali-Bashi M, Rezaei Z, Dehghanian F, Rezaei H (2015). Multiplex PCR based screening for micro/partial deletions in the AZF region of Y-chromosome in severe oligozoospermic and azoospermic infertile men in Iran. Iran J Reprod Med.

[32] Tiepolo L, Zuffardi O (1976). Localization of factors controlling spermatogenesis in the nonfluorescent portion of the human Y chromosome long arm. Hum Genet.

[33] Yu XW, Wei ZT, Jiang YT, Zhang SL (2015). Y chromosome azoospermia factor region microdeletions and transmission characteristics in azoospermic and severe oligozoospermic patients. Int J Clin Exp Med.

[34] Hopps CV, Mielnik A, Goldstein M, Palermo GD, Rosenwaks Z, Schlegel PN (2003). Detection of sperm in men with Y chromosome microdeletions of the AZFa, AZFb and AZFc regions. Hum Reprod.

[35] Burgoyne PS, Mahadevaiah SK, Sutcliffe MJ, Palmer SJ (1992). Fertility in mice requires X-Y pairing and a Y-chromosomal "spermiogenesis" gene mapping to the long arm. Cell.

[36] Ogata T, Wakui K, Kosho T, Muroya K, Yamanouchi Y, Takano T, Fukushima Y, Rappold G, Suzuki Y (2000). Structural analysis of a rare rearranged Y chromosome and its bearing on genotype-phenotype correlation. Am J Med Genet.

[37] McKee BD, Wilhelm K, Merrill C, Ren X (1998). Male sterility and meiotic drive associated with sex chromosome rearrangements in Drosophila. Role of X-Y pairing. Genetics.

